# Multiscale Residual Weighted Classification Network for Human Activity Recognition in Microwave Radar

**DOI:** 10.3390/s25010197

**Published:** 2025-01-01

**Authors:** Yukun Gao, Lin Cao, Zongmin Zhao, Dongfeng Wang, Chong Fu, Yanan Guo

**Affiliations:** 1 School of Information and Communication Engineering, Beijing Information Science and Technology University, Beijing 100101, China; gaoyukun23@bistu.edu.cn (Y.G.); charlin@bistu.edu.cn (L.C.); yananguo@bistu.edu.cn (Y.G.); 2 Key Laboratory of the Ministry of Education for Optoelectronic Measurement Technology and Instrument, Beijing Information Science and Technology University, Beijing 100101, China; 3 Beijing TransMicrowave Technology Company, Beijing 100080, China; wdf@tsmtc.com; 4 School of Computer Science and Engineering, Northeastern University, Shenyang 110169, China; fuchong@mail.neu.edu.cn

**Keywords:** deep learning (DL), human activity recognition (HAR), contrastive learning, radar micro-Doppler signatures, time-Doppler images

## Abstract

Human activity recognition by radar sensors plays an important role in healthcare and smart homes. However, labeling a large number of radar datasets is difficult and time-consuming, and it is difficult for models trained on insufficient labeled data to obtain exact classification results. In this paper, we propose a multiscale residual weighted classification network with large-scale, medium-scale, and small-scale residual networks. Firstly, an MRW image encoder is used to extract salient feature representations from all time-Doppler images through contrastive learning. This can extract the representative vector of each image and also obtain the pre-training parameters of the MRW image encoder. During the pre-training process, large-scale residual networks, medium-scale residual networks, and small-scale residual networks are used to extract global information, texture information, and semantic information, respectively. Moreover, the time–channel weighting mechanism can allocate weights to important time and channel dimensions to achieve more effective extraction of feature information. The model parameters obtained from pre-training are frozen, and the classifier is added to the backend. Finally, the classifier is fine-tuned using a small amount of labeled data. In addition, we constructed a new dataset with eight dangerous activities. The proposed MRW-CN model was trained on this dataset and achieved a classification accuracy of 96.9%. We demonstrated that our method achieves state-of-the-art performance. The ablation analysis also demonstrated the role of multi-scale convolutional kernels and time–channel weighting mechanisms in classification.

## 1. Introduction

In recent years, human activity recognition (HAR) has attracted widespread attention. HAR has broad application potential in healthcare [1], smart homes [2], traffic pedestrian safety [3], national border security [4], and rehabilitation [5]. The purpose of HAR is to identify the activities a person performs with the help of sensors in order to allow computing systems to proactively provide assistance to the users [6]. Deep learning (DL) has developed rapidly and can automatically extract hierarchical knowledge of high-level features from complex data in large-scale labeled datasets [7]. Gradually, it has also demonstrated excellent performance in HAR tasks.

In HAR, there are a number of wearable sensors (IMUs [8], accelerometers, gyroscopes, etc.) worn at different locations on the human body that can capture sampled activity data of kinematic trajectories, such as the WISDM [9], UCI-HAR [10], MHealth [11], and PAMAP2 [12] datasets, which are one-dimensional time series. Recognition of human activities can be achieved by classifying these one-dimensional datasets with features. Nidhi Dua et al. [10] proposed a multi-input CNN-GRU model that omits a lot of feature engineering and data preprocessing and directly performs automatic feature extraction and classification of activities with improved classification accuracy. A multi-input CNN-BiLSTM model was proposed [9], which is able to learn local features and long-term dependencies in sequential data, automatically extract features, and improve classification robustness. There is also a portion of biomedical signal datasets with electrodes measuring electrical signals on the surface of the body, such as electroencephalograms (EEGs) [13], non-invasive surface electrodes (sEMGs) [14], and electrodes measuring electrical signals inside the muscles, such as invasive needle electrodes (iEMGs) [14], which are extracted and categorized, and the patient is monitored in the areas of rehabilitation, diagnosis of diseases, and recognition of activities. In the field of gait analysis [15,16], the goal of a robotics-based assistant is to replicate a movement pattern that is as close as possible to the normal state in terms of temporal and spatial parameters for application in the clinical assessment and rehabilitation of patients with neurological disorders. The LSTM-CNN sequential model [17] is able to generate gait reference trajectories at different walking speeds, which can be used to provide personalized gait reference trajectories for the rehabilitation of amputees and stroke patients using rehabilitation systems such as exoskeleton robots.

But these sensors are usually uncomfortable for the users and do not provide a long-term solution for activity monitoring. Radar is one of the methods used as a non-contact sensor due to its high stability to light and weather conditions and the protection of visual privacy. The return signal modulated by the target carries rich time-varying signals instead of capturing range and speed information of the target’s visually shaped activity [18]. This makes it more user-friendly. Therefore, radar-based human activity recognition has received widespread attention. Radar-based human activity recognition can be converted into a classification task for images by signal processing the acquired Doppler echoes and visualizing them as time-Doppler images. The different types of datasets collected by different sensors are shown in Table 1.

The cost reduction of collecting radar micro-Doppler signatures has led to radar micro-Doppler technology becoming more rapidly developed. Radar micro-Doppler echoes are caused by the subject’s micro-motor dynamic characteristics [19], which can reflect the change in Doppler frequencies of various parts of a person’s body over time as he or she moves. Time-Doppler images in radar 2D echoes contain enough time-varying micro-Doppler information with rich human behavioral features, which is most important for radar-based HAR. When humans perform different movements, the main Doppler shift is caused by the torso, while micro-Doppler is generated by rotating or vibrating parts, such as the legs, feet, and hands. For different activities, the time-Doppler images are different, and thus, valid HAR can be performed based on different time-Doppler images. There are already many methods that use Doppler, time, and distance dimensions for classification [20,21]. Most existing HAR methods use fully supervised methods, but it is difficult and time-consuming to label large amounts of data in the radar field [22,23]. He et al. [24] used a multiscale residual attention network for activity and person recognition, and the results were significantly improved. It usually leads to poor generalization and overfitting problems, especially when the training data are scarce. It is difficult and time-consuming to label large amounts of data in the radar field, so using sparsely labeled datasets is more beneficial for practical applications. In addition, the time-Doppler images are approximately the same for each human activity feature with small differences, and it is not necessary to label all the radar data samples. However, training deep learning models with a small amount of labeled data often leads to overfitting. Therefore, the performance of HAR methods is often hindered by the amount of labeled data. And most deep learning models do not emphasize the time dimension information in the time-Doppler images, leading to redundancy in the model parameters. How to solve the problem of low classification accuracy with a relatively small amount of labeled data has become one of the main concerns in the field of HAR.

Self-supervised learning is popular due to its ability to avoid the cost of annotating large-scale datasets [25]. Self-supervised learning mainly utilizes pretext tasks to mine its own supervised information from large-scale unsupervised data, and trains the network with this constructed supervised information so that it can learn representations that are valuable for downstream tasks [26]. Contrastive learning is a self-supervised representation learning method based on recognizing similarities and differences between images. The goal is to obtain closer feature representations from positive samples (similar samples) and separate feature representations from negative samples (dissimilar samples). It can be viewed as a specific type of pretext task, where sparsely labeled data are used to fine-tune a pre-trained model of feature representations for a wide range of downstream classification tasks. Contrastive learning has recently become a dominant component of self-supervised learning in computer vision (CV), natural language processing (NLP), and other fields [27]. These methods have shown superior performance in visual representation learning and are less limited in the number of labels on the datasets.

The backbone network of most self-supervised contrastive learning methods are small convolutional kernels which have smaller receptive fields (ERFs). However, increasing the effective receptive fields in large kernel designs is crucial in many downstream tasks [28]. And traditional small convolutional kernel CNNs tend to extract texture information from images [29], but time-Doppler images are mainly based on global shape information clues of time series, rather than texture information. The shape information of Doppler changes in the time dimension can reflect the different activities of the human body. But most CNN networks are not sensitive to time series features. Expanding the kernel size of CNNs can effectively focus on shape-based global information in image recognition [30]. Therefore, when classifying time-Doppler images, the convolutional kernel should be moderately increased and attention should be paid to the global shape information of the time series.

In this paper, we propose a new self-supervised contrastive learning algorithm, the multiscale residual weight classification network with contrastive learning. The MRW-CN includes a multiscale residual weight (MRW) image encoder and a classifier. Specifically, we pre-train the MRW image encoder with unlabeled data and apply a self-supervised contrastive learning method to extract the feature representation information of each image. Next, the classifier is fine-tuned with a small number of labeled datasets to handle the downstream behavior recognition classification task.

The contributions of this paper can be summarized as follows:

(1) We collected datasets for eight dangerous activities including “walking”, “squatting”, “standing up”, “picking up”, “drinking”, “ falling”, “stabbing forward”, and “shoving”.

(2) The MRW-CN includes a multiscale residual weight (MRW) image encoder and a classifier. The MRW image encoder is divided into three distinctive residual networks. Large kernels can effectively extract global information. And the time–channel weighting mechanism effectively extract more important time segments in the channel.

(3) In order to evaluate the performance of the algorithm, we used our measured dataset for validation. The experimental results showed that the recognition accuracy was 96.9% with fine-tuning using only 10% of the labeled data, and the highest F1-score was 0.99. And we found that contrastive learning gained more benefits from larger batch sizes and longer training. Ablation analysis demonstrated the efficiency of the residual networks with multiscale convolutional kernels and the time–channel weighting mechanism. It showed that the MRW-CN model is stable and robust.

The remainder of this paper is organized as follows: Section 2 reviews the research on HAR and contrastive learning. In Section 3, multiscale residual weight classification network with contrastive learning is described in detail. In Section 4, the activities and scenarios of our collected dataset are presented. The experimental results and some discussions are presented in Section 5. Finally, a conclusion is drawn in Section 6.

## 2. Related Work

Human activity recognition (HAR) is mostly implemented with fully supervised training. However, it is time-consuming and laborious due to labeling a large number of radar signal instances. Self-supervised contrastive learning techniques have developed rapidly in recent years to use sparsely labeled data for training, and have been actively investigated due to their similarity to supervised methods and good results.

In recent years, researchers have been utilizing radar echoes for feature extraction as a means to facilitate classification tasks. TD images in 2D radar echoes are more often used for classification to reduce the budget complexity under the condition of guaranteed classification accuracy. Micro-Doppler datasets of different frequencies, bandwidths, and waveforms have also been collected for training in various fields [31], and dual-labeled data of human activity recognition and person identity are also available [24]. Yang et al. [32] proposed an angle-insensitive classifier for omnidirectional classification problems, which can achieve behavior recognition at various angles. Kim et al. [33] proposed a method for estimating the position and velocity of targets by fusing Doppler data from multiple sensors. HAR-based radar has low power consumption and can extract target features even in harsh weather conditions around the clock. It can be applied to data collection through walls, so radar-based HAR is becoming increasingly popular. To solve the problem of angular sensitivity in micro-Doppler classification, Erol et al. [34] used adversarial learning methods to generate data from different environments, and using PCA for filtering, the trained CNN network effectively improved accuracy. Mehmet et al. [35] used unsupervised pre-trained deep convolutional encoders. Park et al. [36] proposed using pre-training with RGB images and fine-tuning with radar datasets. Gurbuz et al. [31] used generative adversarial networks to generate synthetic datasets for pre-training to recognize activity. Taylor et al. [37] employed component analysis to expand datasets effectively to improve the recognition accuracy of CNN models. This has been proven to be more effective than other models. Qiao et al. [1] used the CPCAN model, which can separate the micro-Doppler signals between the human trunk and limbs to achieve efficient training. However, almost all of these methods require a large amount of labeled data. It is a challenging task to perform effective feature extraction and perform classification when there is little data labeling. Li et al. [38] proposed “joint domain and semantic transfer learning” (JDS-TL), which achieved good classification results. Du et al. [39] fine-tuned the pre-trained network on ImageNet, achieving higher accuracy in shorter training epochs.

Contrastive learning focuses on mining its own feature information from large-scale data, and training the network with this constructed supervised information so that it can learn representations that are valuable for downstream tasks. Self-supervised learning, as a form of contrastive learning, is performed by comparing the difference in distance between positive and negative samples. It is popular because it does not require manual labeling. It can learn general image and video features from large-scale unlabeled data to handle a large number of downstream tasks in image classification, target detection, activity recognition, etc. Hadsell et al. [40] first proposed learning representations by comparing positive and negative pairs in 2006. Then, Dosovitskiy et al. [41] proposed the consideration of each instance as a class represented by a feature vector. Eldele et al. [42] proposed TS-TCC for learning representations in unlabeled data, which is equivalent to fully supervised training. Since then, self-supervised methods have been proposed for classification tasks. Chen et al. [43] proposed a generalized SSL embedding network that learns the characteristics of the data themselves to serve downstream tasks. Sarkar et al. [44] proposed a self-supervised multi-task learning framework with fine-tuning of the dense layer. Chen et al. [45] proposed a network that can be trained with a small amount of labeled data for effective classification. Grill et al. [46] proposed a network which can represent learning from a picture under different enhanced views without negative examples. To avoid collapsing solutions in self-supervised learning, Xinlei et al. [47] proposed siamese networks. Chen et al. [48] proposed large networks in the pre-training and fine-tuning phases; the fewer the labels, the more the network gains. Tang et al. [49] explored the possibilities of the SimCLR method in HAR and mentioned that random rotation of images yielded better results for HAR.

The purpose of this article is to design an effective contrastive learning network that uses a small labeled dataset to achieve classification tasks. The main challenge here is how to design a network model to more effectively learn the shape differences of time series in time-Doppler images features.

## 3. The Multiscale Residual Weight Classification Network

**Figure 1:**
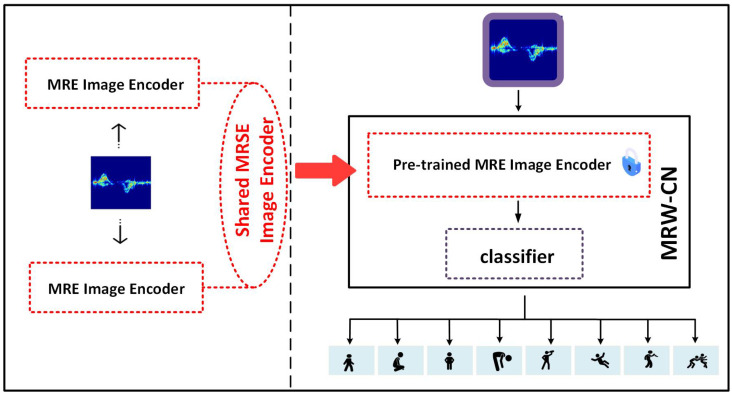
The overall flow of the MEW-CN model.

In this paper, we propose the multiscale residual weight classification network (MRW-CN) with contrastive learning to achieve human activity recognition. The overall flow of the MEW-CN model is shown in Figure 1. It contains a multiscale residual weight (MRW) image encoder and a classifier. The MRW image encoder is categorized into three different residual networks, namely the large-scale residual network, medium-scale residual network, and small-scale residual network. The classifier consists of two fully connected layers. Firstly, all the unlabeled radar time-Doppler images are pre-trained by contrastive learning with an MRW image encoder to extract representative vectors on all radar time-Doppler images. The large-scale network extracts more global information, and the medium-scale residual network extracts more texture information. The small-scale residual network extracts more semantic information. The three TC weighting mechanisms can effectively extract the important feature fragments in the two dimensions of channel and time. The last two convolutional blocks extract the salient information. Secondly, the pre-trained MRW image encoder parameters are retained and the classifier is added at its end. Finally, by freezing the parameters of the pre-trained MRW image encoder and fine-tuning the classifier using a small amount of labeled data, effective classification of the radar dataset is achieved. In this section, the composition and training process of MRW-CN, the preprocess of contrastive learning by MRW image encoder, and the specific structure of the MRW image encoder will be described in detail.

### 3.1. Specific Structure of the MRW Image Encoder

**Figure 2:**
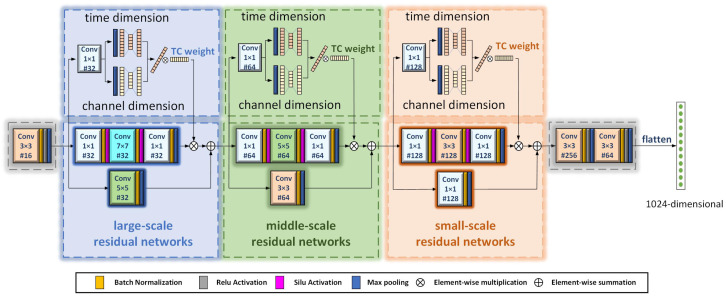
Details of the multiscale residual weight (MRW) image encoder structure. #16 represents a total of 16 channel layers. 3 × 3 represents a convolution kernel of 3 × 3.

In order to extract the salient feature representations between image pairs more efficiently for downstream classification tasks, we designed the MRW image encoder. The MRW image encoder mainly consists of large-scale residual networks, medium-scale residual networks, and small-scale residual networks. Its specific structure is shown in Figure 2.

The first part of the MRW image encoder is a 3 × 3 convolutional kernel block to extract the overall features in the image $z_{i}$. A BN layer, activation function, and max pooling are applied after the convolution operation. The BN layer accelerates network convergence, and the max pooling layer reduces computational complexity.

(1) $$F_{o}\left(z_{i}\right)=con_{3}\left(z_{i}\right)$$

The second part of the MRW image encoder consists of the three different multiscale residual networks. The first residual network is a large-scale residual network. The main branch is a 7 × 7 convolutional kernel block which extracts the broader global information from the overall features. The two 1 × 1 kernel convolutions are used to fuse the features in each channel to increase the nonlinearity of the information interaction. Each convolution operation is followed by the addition of a BN layer, an activation function, and maximum pooling. In addition, we apply the Silu activation function between two convolutional blocks. Compared with the ReLU activation function, the SiLU function has a smoother curve near zero and can retain more information [50]. The residual branch of the large-scale residual network is a 5 × 5 kernel convolution block to extract more complete global information while solving the gradient vanishing and gradient explosion problems.

For time–Doppler images, the time dimension information is particularly important. We have incorporated the time–channel weighting mechanism into all three types of residual networks. In the TC weighting mechanism, the maximum pooling is first performed along the time dimension and channel layer dimension, so that each layer obtains one data point. Then, the weights are obtained through a two-layer fully connected transformation. The two-dimensional data of the time layer and channel layer are reduced by 1/8 and then expanded by 8 times, respectively. The obtained results have the same time dimension and channel layer size as the original data. Weighting coefficients are yielded with respect to the time and channel dimensions. The data block and extracts are multiplied based on the importance of the data. This focuses not only on the more important channel dimensions, but also on finding important time periods for each time–Doppler image. The TC weight matrix obtained at the large-scale residual network is 56 × 32.

(2) $$F_{g}\left(z_{i}\right)=\left[TC(con_{1,7,1}\left(F_{o}\left(z_{i}\right)\right))\right]+con_{5}\left(F_{o}\left(z_{i}\right)\right)$$

Afterwards, the second residual network is a medium-scale residual network. The main branch is a 5 × 5 kernel convolution, which extracts more texture information from the global information, and the convolution of the two 1 × 1 kernel increases the nonlinearity of the model. This time–channel weighting allows for more focus on the more important time areas and channel layers of the texture information. The TC weight matrix obtained at the medium-scale residual network is 28 × 64. Its residual branch is a 3 × 3 kernel convolution block to extract more complete texture information.

(3) $$F_{t}\left(z_{i}\right)=\left[TC(con_{1,5,1}\left(F_{g}\left(z_{i}\right)\right))\right]+con_{3}\left(F_{g}\left(z_{i}\right)\right)$$

The third residual network is a small-scale residual network. The main branch is a 3 × 3 kernel convolution block that adds the ability to extract complex semantic information from texture information. The two 1 × 1 kernel convolution blocks increase the nonlinearity of the network. This time–channel weighting allows for more focus on the more important time periods and channel layers of the semantic information. The TC weight matrix obtained at the small-scale residual network is 14 × 128. Its residual branch is a 1 × 1 kernel convolution block for more completeness in extracting semantic information.

(4) $$F_{s}\left(z_{i}\right)=\left[TC(con_{1,3,1}\left(F_{t}\left(z_{i}\right)\right))\right]+con_{1}\left(F_{t}\left(z_{i}\right)\right)$$

To extract salient information from the image, we use two 3 × 3 kernel convolution blocks in the last part of the MRW image encoder. It integrates the extracted image semantic information to obtain the salient features ${\hat{z}}_{i}$ of each image. The salient features of all the augmented image pairs are finally obtained and flatten to obtain 1024-dimensional features that can be used for downstream classification tasks.

(5) $${\hat{z}}_{i}=E\left(z_{2i}\right)=con_{3}\left(con_{3}\left(F_{s}\left(z_{i}\right)\right)\right)$$

The time–Doppler images to be classified are directly input into the pre-trained MRW image encoder, which undergoes the extraction of overall features, global features, texture features, and semantic features, and finally integrates them to obtain 1024-dimensional salient features.

### 3.2. MRW Image Encoder Pre-Training Process

**Figure 3:**
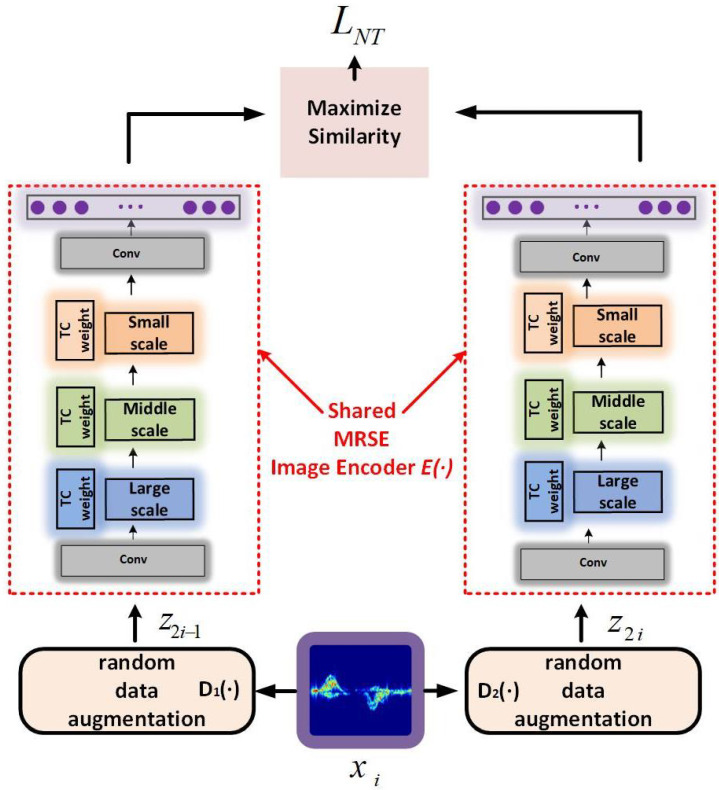
Contrastive learning process of the MRW image encoder. The two MRW image encoder parameters are shared.

We use the MRW image encoder to perform feature extraction on unlabeled radar time–Doppler images by contrastive learning pre-training, and its parameters are fixed to handle downstream classification tasks. The specific pre-training process is presented in Figure 3.

Firstly, two data augmentation operations $D_{1}\left(•\right)$ and $D_{2}\left(•\right)$ are applied to all images $x_{i}$$\left(i\in n_{t}\right)$ to obtain two different views $z_{2i-1}$ and $z_{2i}$, where $n_{t}$ denotes the amount of data used for training.

(6) $$z_{2i-1}=D_{1}\left(x_{i}\right)$$

(7) $$z_{2i}=D_{2}\left(x_{i}\right)$$

The two data augmentation operations are a collection of random cropping, random rotation, random color distortion, and random Gaussian blurring operations. The two different views $z_{2i-1}$ and $z_{2i}$ are an image pair. The two views from the same image are treated as positive examples and the two views from different images are treated as negative examples.

Secondly, all augmented image pairs are separately input into the MRW image encoder $E\left(•\right)$ with shared parameters to obtain the salient feature representations ${\hat{z}}_{2i-1}$ and ${\hat{z}}_{2i}$ of all augmented image pairs. The feature representation of each augmented view consists of 1024-dimensional vectors.

(8) $${\hat{z}}_{2i-1}=E\left(z_{2i-1}\right)$$

(9) $${\hat{z}}_{2i}=E\left(z_{2i}\right)$$

Finally, the feature representation vectors ${\hat{z}}_{2i-1}$ and ${\hat{z}}_{2i}$ learned by the MRW image encoder are extracted. By the contrastive learning approach, the network parameters of the MRW image encoder are trained. The normalized temperature-scaled cross-entropy loss function is shown as follows:

(10) $$l\left(i,j\right)=-\log\frac{\exp(sim\left({\hat{z}}_{i},{\hat{z}}_{j}\right)/\tau )}{\sum_{k=1}^{2N}I_{\left[k\neq i\right]}\exp\left(sim\left({\hat{z}}_{i},{\hat{z}}_{k}\right)/\tau \right)}$$

(11) $$sim\left(u,v\right)=\frac{u^{T}v}{∥u∥∥v∥}$$

where $sim\left(u,v\right)$ is the cosine similarity, which denotes the dot product between the standardized *u* and *v*. I is an indicator function evaluating to 1 iff k ≠ i. The loss is calculated for all image pairs. τ is the temperature parameter, which determines the amount of salient information extracted within the class. The temperature parameter τ is set to 0.5 in the loss function l(i,j). The numerator indicates the similarity between samples within the same batch and their corresponding positive samples. The numerator encourages greater similarity between the positive samples. The denominator represents the similarity between samples within the same batch and all their corresponding negative samples. The denominator encourages less similarity between negative samples.

### 3.3. MRW-CN Method

**Figure 4:**
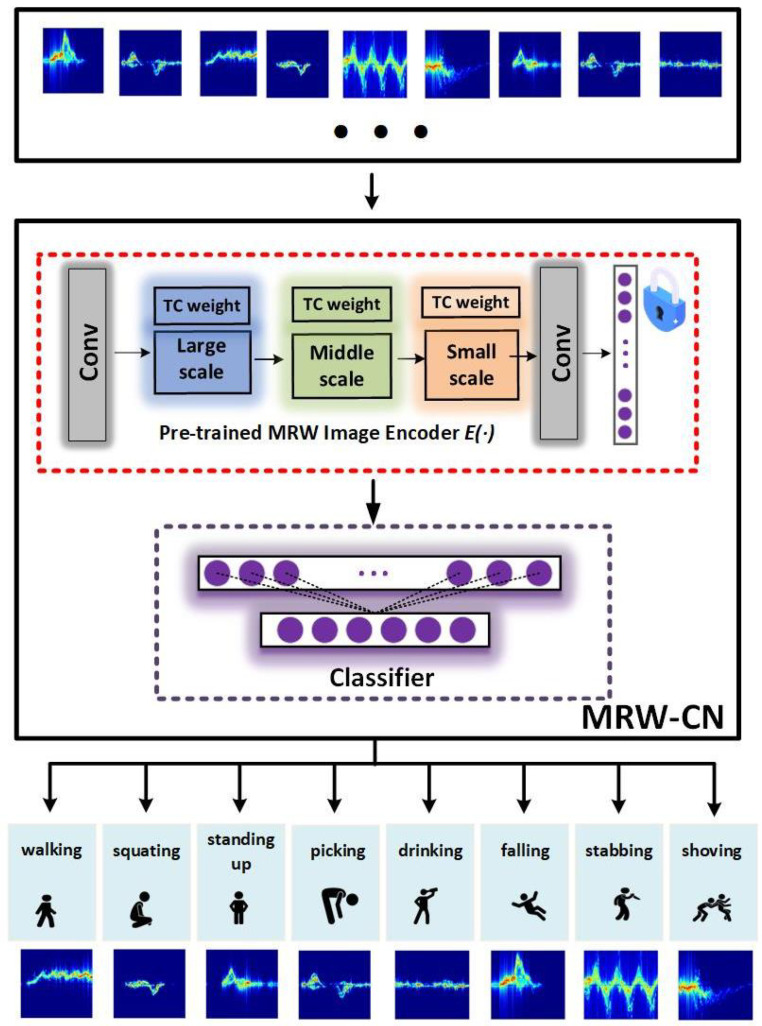
The MRW-CN method contains the MRW image encoder and classifier.

In the radar time–Doppler images, each pixel point contains information in both the time and Doppler dimensions.

(12) $$X=\left\{\left(x_{i},y_{i}\right),i=1,\cdots ,n\right\}$$

where $y_{i}$ denotes the *i*th label corresponding to the *i*th radar image $x_{i}$. *y* represents the eight activities on the dataset. The dataset is used for the downstream classification task. The purpose of our model is to train a deep learning model to classify the time–Doppler images and identify the corresponding activities in dataset *X*. It is usually difficult to achieve satisfactory performance if the deep learning model is trained directly with a small amount of data. The specific structure of the MRW-CN is shown in Figure 4.

For better classification of time–Doppler images, we proposed the MRW-CN network. The MRW-CN consists of two parts, the pre-trained MRW image encoder E(•) and the classifier C(•). The MRW image encoder E(•) is a multiscale residual weighted network consisting of large-scale, medium-scale, and small-scale residual networks. The parameters of the MRW image encoder are obtained by pre-training with contrastive learning. The classifier C(•) is composed of two fully connected layers and the ReLU activation function. The two fully connected layers have dimensions of 512 and 8, respectively.

(13) C(•)=FC(σ(FC(•)))

(14) $${\hat{y}}_{i}=soft\max\left(C\left(E\left(x_{i}\right)\right)\right)$$

where σ represents the ReLU activation function. ${\hat{y}}_{i}$ is the predicted label of the *i*th sample. The softmax function is used after the classifier to predict the labels ${\hat{y}}_{i}$ of the salient features obtained by the pre-trained MRW image encoder.

The pseudocode of the training process of MRW-CN is depicted in Algorithm 1.
**Algorithm 1** Training process.**Input:** 
TD images;**Output:** 
Label: 0–5 (eight types of activities);  1:**for** all $i\in \left\{1,\cdots ,N\right\}$ **do**  2:   # the first augmentation  3:   $z_{2i-1}=D_{1}\left(x_{i}\right)$  4:   Extract $F_{o}$, $F_{g}$, $F_{t}$, $F_{s}$, ${\hat{z}}_{2i-1}=E\left(z_{2i-1}\right)$  5:   # the second augmentation  6:   $z_{2i}=D_{2}\left(x_{i}\right)$  7:   Extract $F_{o}$, $F_{g}$, $F_{t}$, $F_{s}$, ${\hat{z}}_{2i}=E\left(z_{2i}\right)$  8:**end for**  9:Minimize $L_{NT}$ to train the MRW image encoder *E* by contrastive learning;10:Freeze the parameters of the MRW image encoder *E* and train the classification network by minimizing *L*;11:Save the MRW-CN model.

## 4. Dataset Description

**Figure 5:**
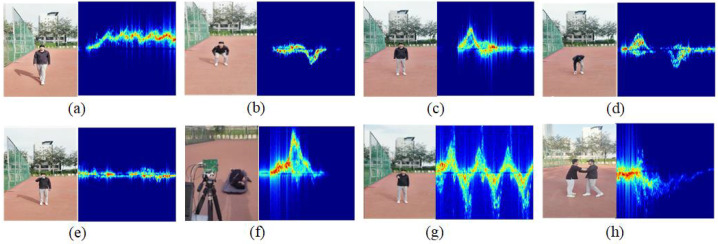
Scenes from our collected datasets and some typical radar time–Doppler images. (**a**) Walking (A1). (**b**) Squatting (A2). (**c**) Standing up (A3). (**d**) Picking up (A4). (**e**) Drinking (A5). (**f**) Falling (A6). (**g**) Stabbing forward (A7). (**h**) Shoving (A8).

To evaluate the performance of the proposed MRW-CN, we used the AWR1642 microchip from TEXAS INSTRUMENTS to collect human activity recognition datasets outdoors for validation. Most of the human activity recognition datasets focus on daily activities. To increase the breadth of the application, we collected a number of dangerous activities. The datasets included eight activities, namely, “walking”, “squatting”, “standing up”, “picking up”, “drinking”, “ falling”, “stabbing forward”, and “shoving”. There were a total of 20 volunteers around 25 years old. There were a total of 20 volunteers around 25 years old. Among them, there were five people with a height of 170 cm and a weight of 135 kg. There were 10 people with a height of 175–180 cm and a weight of 140 kg. There were five people with a height of 180–185 cm. Among them, two people weighed 145 kg and three people weighed 150 kg. The testing time was 5 s. The human body was 5 m away from the radar when making movements. The “walking” movement involved moving from 5 m to 3 m away from the radar. Each activity was repeated 10 times, with 200 sets of data for each activity. There were a total of 1600 time–Doppler images for all eight activity classes. The onboard radar antenna operated in the 76 GHz to 81 GHz millimeter wave range. By utilizing infinite impulse response (IIR) filters and short-time Fourier transform (STFT) conversion, micro-Doppler feature images of different people performing different activities were obtained. The HAR problem could be solved as an image classification task due to the large differences in features between different activities. A few typical radar time–Doppler images from the our self-collected dataset are shown in Figure 5.

The horizontal coordinate of the time–Doppler images is the time dimension, and the vertical coordinate represents the micro-Doppler characteristics of the human body during different activities, which are the trajectory characteristics of the activities. Due to the large differences in features between different activities, different samples of time–Doppler images under different activities are also more different from each other. The characteristics of the same activity are similar, and the different samples of the time–Doppler images under the same activity are also more similar. We resized the time–Doppler images to 224 × 224 pixels and input them into the designed MRW-CN model.

## 5. Experimental Results and Discussion

### 5.1. Training Process

#### 5.1.1. Training of MRW Image Encoder

In the contrastive learning pre-training process used for feature extraction in the MRW image encoder, all image pairs were fed into the MRW image decoder. This step sequentially extracted the overall features, global features, texture features, and semantic features. Finally, integration and merging were performed to obtain 1024-dimensional salient features ${\hat{z}}_{2i-1}$ and ${\hat{z}}_{2i-1}$. The loss function used was the normalized temperature normalized cross-entropy function $L_{NT}$. The similarity between positive examples of features was maximized and the similarity between negative examples was minimized.

(15) $$L_{NT}=\frac{1}{2N}\sum_{k=1}^{N}\left[l(2k-1,2k)+l(2k,2k-1)\right]$$

where *N* represents the training batch sizes. $L_{NT}$ was used to calculate the sum of the loss of all image pairs and average them. In the pre-training process of contrastive learning for MRW image encoder feature extraction, epochs were set to 200 and the batch size was 256. An adaptive moment estimation (Adam) optimization algorithm was used with the learning rate set to 0.0005.

**Figure 6:**
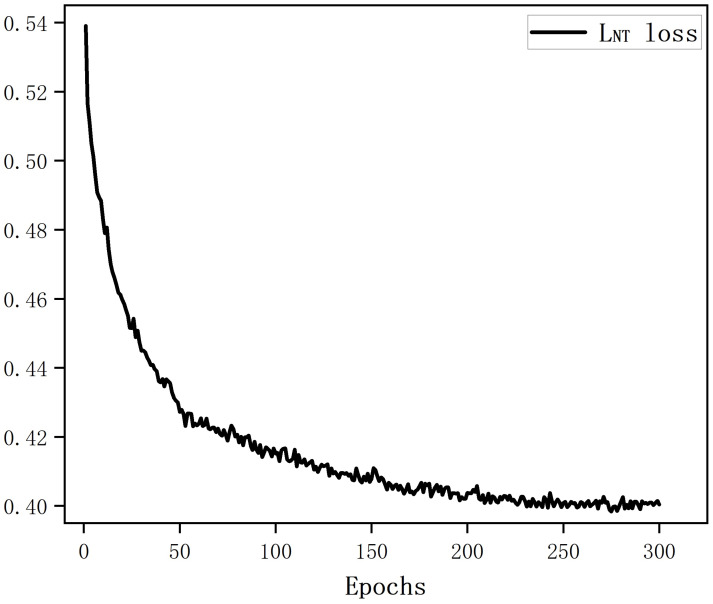
The training loss of the MRW image encoder.

The loss curve of the MRW image encoder is shown in Figure 6. It can be seen that after 200 epochs, the loss remained stable.

#### 5.1.2. Training of the MRW-CN

**Figure 7:**
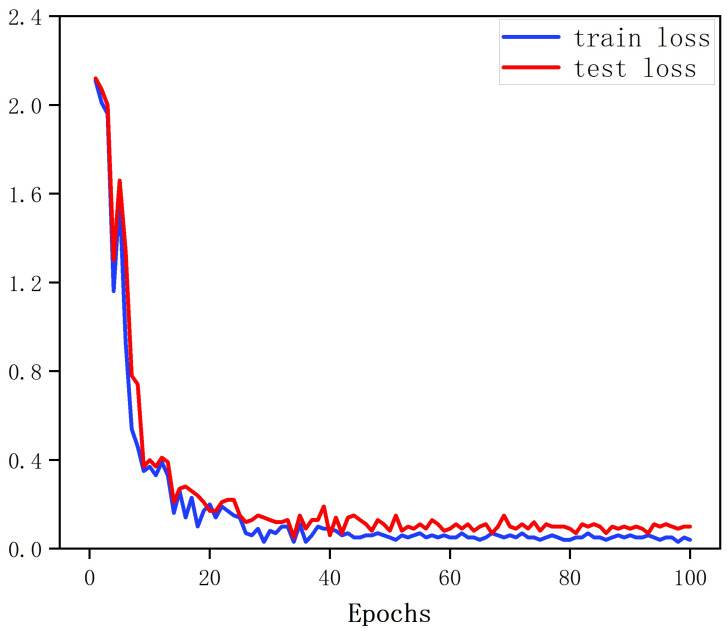
The training loss and the testing loss of the MRW-CN.

When training MRW-CN, the MRW image encoder parameters were locked and only the classifier was fine-tuned. The MRW image encoder obtained salient information from all images. The obtained 1024-dimensional salient features were used to output the classification probabilities of the eight activities through two fully connected layers and a softmax function. The parameters of the classifier were fine-tuned using the cross-entropy loss *L*. The cross-entropy loss function, as shown in (32), was used to train the classifier.

(16) $$L=-\sum_{c=1}^{C_{t}}{\hat{y}}_{i,c}\log\left(y_{i,c}\right)$$

where $C_{t}$ is the total number of activity classes. The MRW-CN model was trained by minimizing the cross-entropy loss. The batch size was set to 16. An adaptive moment estimation (Adam) optimization algorithm was used with a learning rate of 0.0005. The training loss curve for 10% of the labeled data and the test loss function for the test of the data are shown in Figure 7. After about 50 epochs, the two loss curves stabilized.

### 5.2. Classification Results

We used a total of 1671 images together for the contrastive learning self-supervised training of the MRW image encoder. A total of 167 images were selected for training MRW-CN and 1071 images were used for testing. Four types of measurements, namely, True Negatives (TNs), False Positives (FPs), False Negatives (FNs), and True Positives (TPs), are often utilized for assessing the performance of classification results. The four key metrics, accuracy, precision, recall, and F1-score for HAR were calculated with TN, FN, TP, and FP.

(17) $$Accuracy=\frac{TN+TP}{TN+TP+FN+FP}$$

(18) $$Precision=\frac{TP}{TP+FP}$$

(19) $$Recall=\frac{TP}{TP+FN}$$

(20) $$F1\text{-}Score=2*\frac{Recall*Precision}{Recall+Precision}$$

where F1-score is an indicator designed to comprehensively consider precision and recall. We chose to use accuracy and F1-scores as the primary measures.

Since 10% of the labeled radar images were randomly selected, the classification performance fluctuated with the distribution of the selected images. Therefore, we used independent repetitive experiments to obtain the maximum accuracy. Specifically, we selected 10% of the labeled dataset for fine-tuning.

In this study, four evaluation metrics, including accuracy, recall, precision, and F1-score, were used to evaluate the performance of the proposed model. All experimental results were obtained by testing on the same dataset.

**Figure 8:**
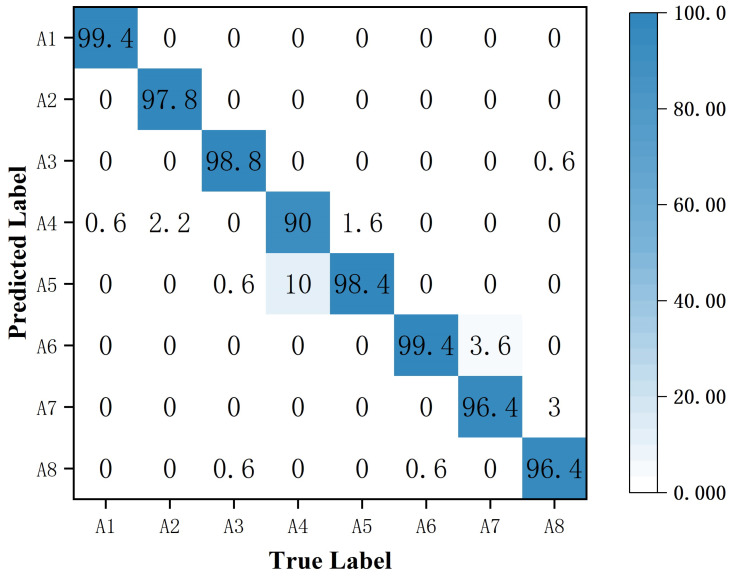
The confusion matrix for the eight types of activities when trained with 10% of the data.

Figure 8 shows the confusion matrix for human activity recognition with 10% of the labeled data. The average classification accuracy using the proposed MRW-CN was 96.9%. “Walking” and “falling” were recognized with the highest accuracy, both at 99.6%. It indicates that the time–Doppler images of “walking” and “falling” are more discriminative than other activities. Since both “picking” and “drinking” have radar Doppler echoes from the bottom to the top of the arm, they are more difficult to distinguish and less accurate. “Picking” had the lowest accuracy compared to the others because the longitudinal and transverse angles between radars changed dynamically when different people bent. It achieved a recognition accuracy of 90%. Therefore, the resulting MD signature was variable and more difficult to identify.

**Figure 9:**
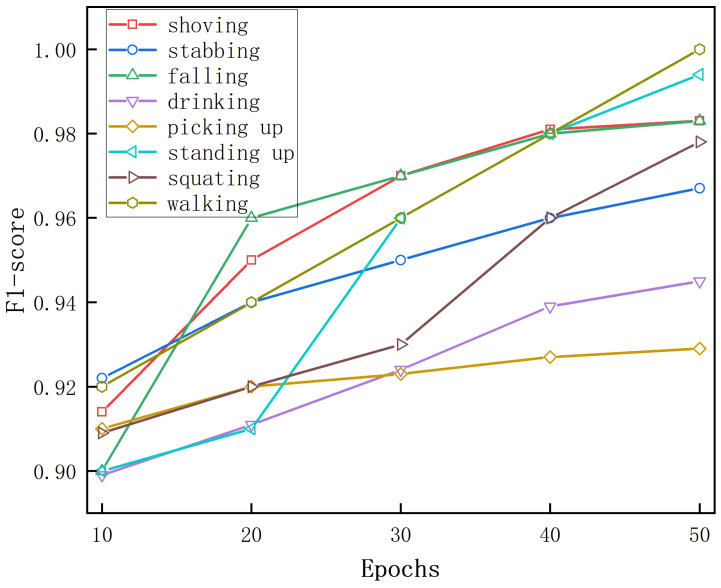
The F1-score curves during training for the eight types of human activity recognition when trained with 10% of the data.

The F1-score curves for eight activities when using 10% of the labeled data are shown in Figure 9. The “walking” activity had the highest F1-score, indicating that the spectrum of “walking” was the most effective for human recognition among the five activities. We investigated the performance of the recognition activities separately. The spectrum for “walking” was the most effective for personal identification, with an F1-score of 0.99, suggesting that the “walking” activity may retain more personal information than its counterparts. The four movements “walking”, “falling”, “shoving”, and“standing up” were different, with F1-scores above 0.92. In contrast, the F1-score for the “picking” spectrogram for activity recognition was the lowest, at about 0.93. The time–Doppler images of the two movements of “drinking” and “picking up” were similar, so their MD signatures were not as distinct as those of the other movements.

### 5.3. Contrastive Learning Gains More Benefits from Larger Batch Sizes and Longer Training

**Figure 10:**
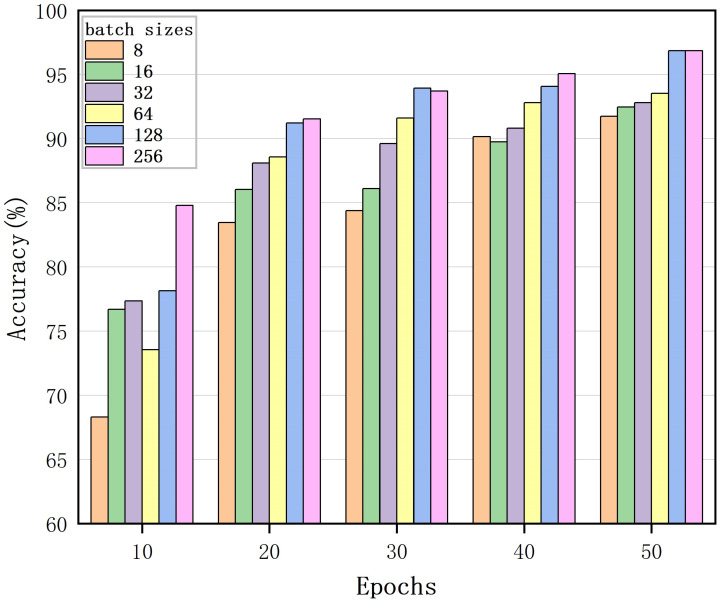
The MRW-CN training process involved utilizing different batch sizes and epochs.

As training stability was achieved after 50 epochs, we only selected the first 50 training epochs for comparison. Figure 10 shows the impact of image batch size on the accuracy of downstream classification tasks when training an MRW image encoder. The larger the batch size value, the higher the final recognition accuracy. When the number of training epochs was larger, larger batch sizes had a significant advantage over smaller batch sizes. With more training epochs, the gap in accuracy between different batch sizes decreased or disappeared. In the contrastive learning of the images, larger batch sizes provided more negative examples and promoted convergence. Longer training also provided more negative examples of images, effectively improving the classification results. The final recognition accuracy was lowest at 83.7% when the batch size was 8. The proposed MRW-CN achieved the best performance with an accuracy 96.9% when the batch size was 256.

### 5.4. Comparison with the State-of-the-Art Methods

To demonstrate the effectiveness of the proposed method for only 10% of the labeled datasets, we compared it with the following state-of-the-art methods:

1. SSTL [51]: This method extracts feature representation, applies pre-training, and uses popular regularization techniques for semisupervised and transfer learning.
2. SimCLR [45]: This method is a visual contrastive learning method using representation vectors between nonlinear transformations.
3. BYOL [46]: This method relies on two neural networks, the online network and the target network. The online network predicts the target network representation of the image in different views.
4. JDS-TL [38]: This method is a semisupervised learning algorithm consisting of two modules—unsupervised adaptive and supervised semantic escape—trained on sparsely labeled datasets.
5. MOCO [52]: This method constructs a dynamic dictionary with a queue and a moving average encoder to improve unsupervised learning performance.
6. SelfHAR [53]: This method combines teacher–student self-training and multi-task self-supervision to learn signal-level representations by predicting distorted versions of inputs.

We also compared the accuracy of the six methods when trained with 5% and 10% of the labeled data. The comparison results are shown in Table 2. It can be seen that the proposed MRW-CN achieved the best performance among these methods with a maximum classification accuracy of 96.9% with only 10% of the labeled data. With a labeled dataset comprising only 5% of the total data, the MRW-CN method achieved an impressive accuracy of 96.3%. This accuracy level also outperformed other existing methods. In addition, we also compared the model parameter count and inference time of these methods. The model complexity (FLOPs) is the number of operations of the model. The computational complexity of the MRW-CN model was the lowest, at 3.8 G. MOCO had the highest computational complexity, at 4.1 G. BYOL had the shortest inference time among all methods, at 2.8 h. The inference time for MRW-CN was 3 h. This indicates that the MRW-CN method can achieve the highest classification accuracy even when the model’s computational complexity is minimized.

### 5.5. Feature Visualization

**Figure 11:**
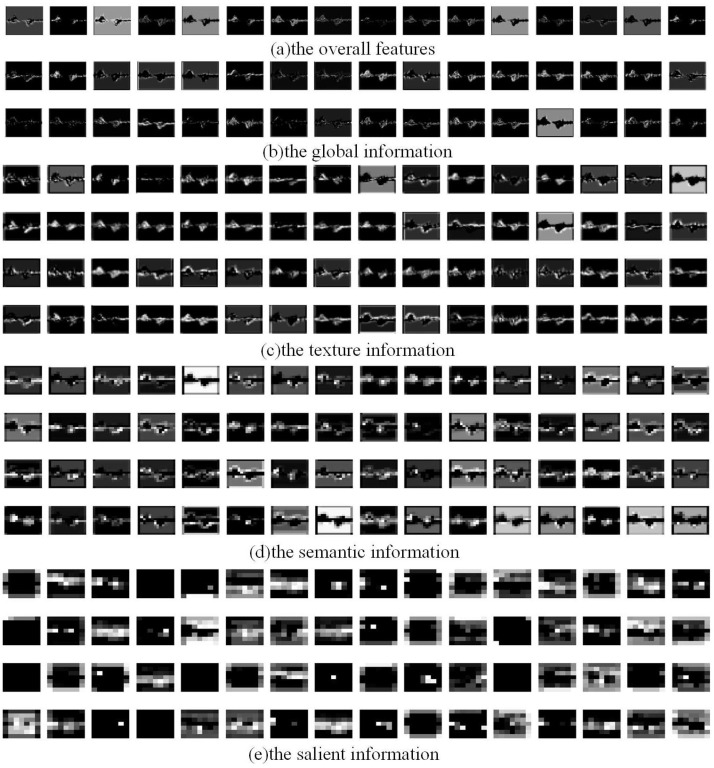
Visualization of features extracted from different parts of the MRW image encoder.

To effectively demonstrate the synergistic effect of different parts of the MRW-CN image encoder, we visualize the extracted features of each part of the model. In part (a) of Figure 11, the overall 16-layer features extracted from the first 3 × 3 convolution are visualized. The overall information mainly highlights the area where the Doppler features are located in the image. The 32 layers of global features extracted from large-scale features are shown in part (b) of Figure 11, reflecting more of the edge changes in the features. In section (c) of Figure 11, the partial texture information extracted by the medium-scale residual network is shown. The texture information not only preserves the edge information, but also extracts the internal texture variation information of features. In section (d) of Figure 11, the partial semantic information extracted by a small-scale residual network is visualized. The semantic information is the deep-level understanding of features by the model. In section (e) of Figure 11, the salient feature representations extracted from the last two convolutional blocks are visualized. The salient feature representations consist of fuzzy information, mainly used to provide effective information in downstream classification tasks. The various parts of the features extracted by the MRW image encoder are different and gradually blurred, indicating its effectiveness in extracting time–Doppler image features.

### 5.6. Effect of the Temperature Parameter τ

**Figure 12:**
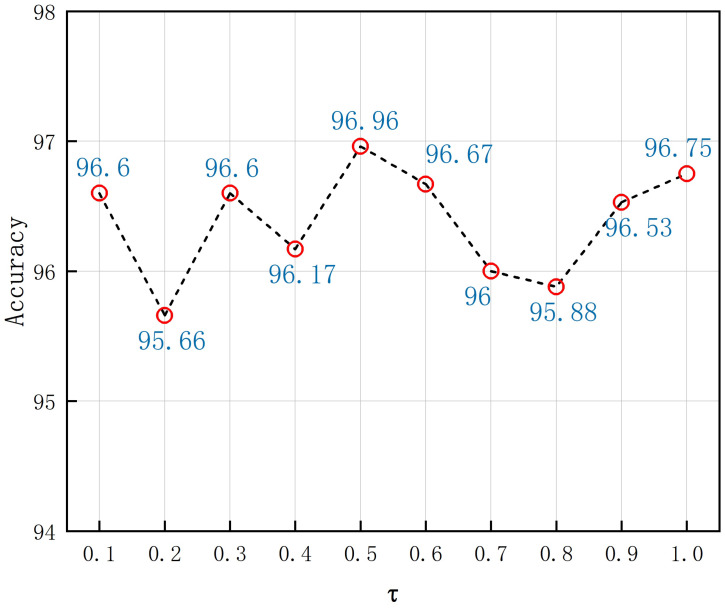
Variations in the maximum recognition accuracy with diverse values of τ.

We performed a sensitivity analysis of the temperature parameter τ in the loss function l(i,j). The performance on the final classification accuracy with different values of τ is shown. The amount of retained salient information within the class varied due to the different values of τ. As can be seen from Figure 12, when the value of τ was set at 0.5, most of the salient information within the class was retained in the input distribution, and the proposed model achieved the best performance with a classification accuracy of 96.9%. When τ was greater than 0.5, increasing the value of the temperature parameter τ led to a noticeable decline in accuracy. When τ was 0.8, the accuracy was 95.88%. When τ was set to 0.2, the least amount of salient information was retained within the class, and the final classification accuracy was only 95.66%. Thus, setting an appropriate value of τ can make the classification task easier while retaining sufficient salient information in the class. In this paper, τ was set to 0.5.

### 5.7. Fully Supervised Training Results

We also explored human activity recognition accuracy when fully supervised training was performed, randomly selecting 1169 images to train MRW-CN and testing with 502 images. The average recognition accuracy of 98.7% was achieved.

**Figure 13:**
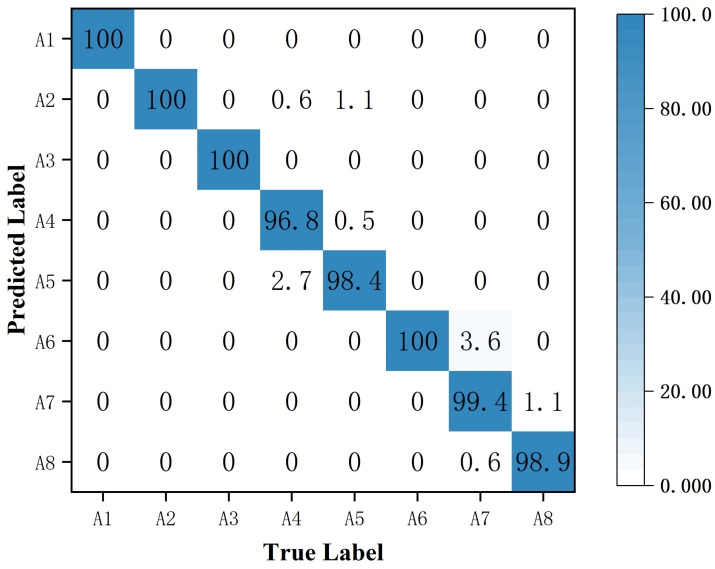
The confusion matrix for eight types of activities in fully supervised training.

The resulting confusion matrix is shown in Figure 13. The recognition accuracy of “walking”, “squatting”, and “standing” was 100%. And the accuracy of “picking” was the lowest, at only 96.8%. It can be seen that our proposed MRW-CN also performed well when performing fully supervised training.

**Figure 14:**
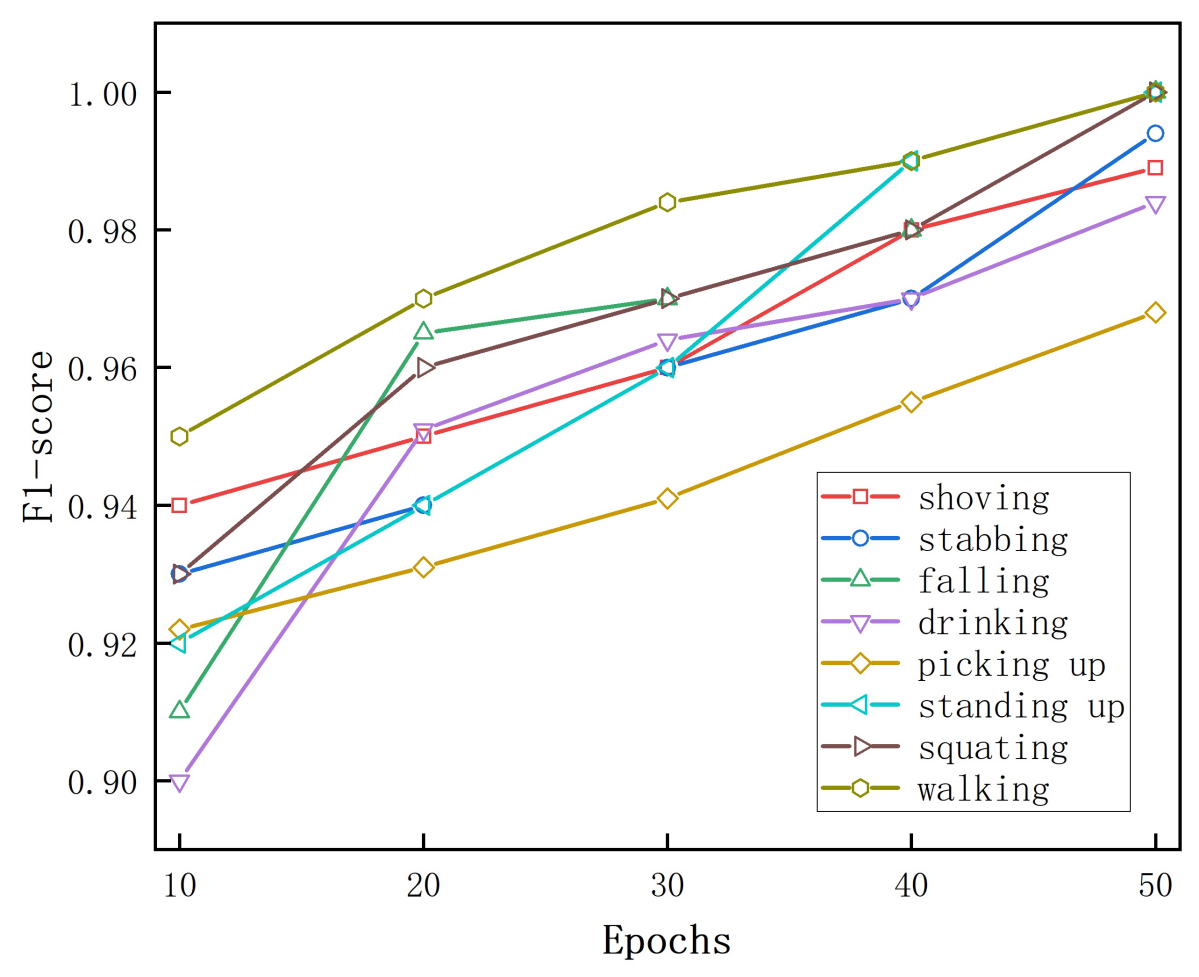
The F1-score curves during training for eight types of activities in fully supervised training.

Figure 14 illustrates how the F1-score of the eight activities grew with the number of training epochs when fully supervised training was performed. At only 10 epochs of training, “walking” had the highest F1-score of 0.95 and “drinking” had the lowest F1-score of 0.9. As the number of training epochs increased, the F1-scores for all eight activities continued to improve. At 50 epochs, both “walking” and “standing” had F1-scores of 1, indicating that these two activities were the easiest to distinguish. “Picking” was more difficult to distinguish, and the F1-score was 0.968.

As can be seen from the above confusion matrix figure and F1-score figure, the MRW-CN is a robust solution for applications when it is trained with full supervision.

### 5.8. The Influence of Convolutional Kernel Size on Classification Accuracy

**Figure 15:**
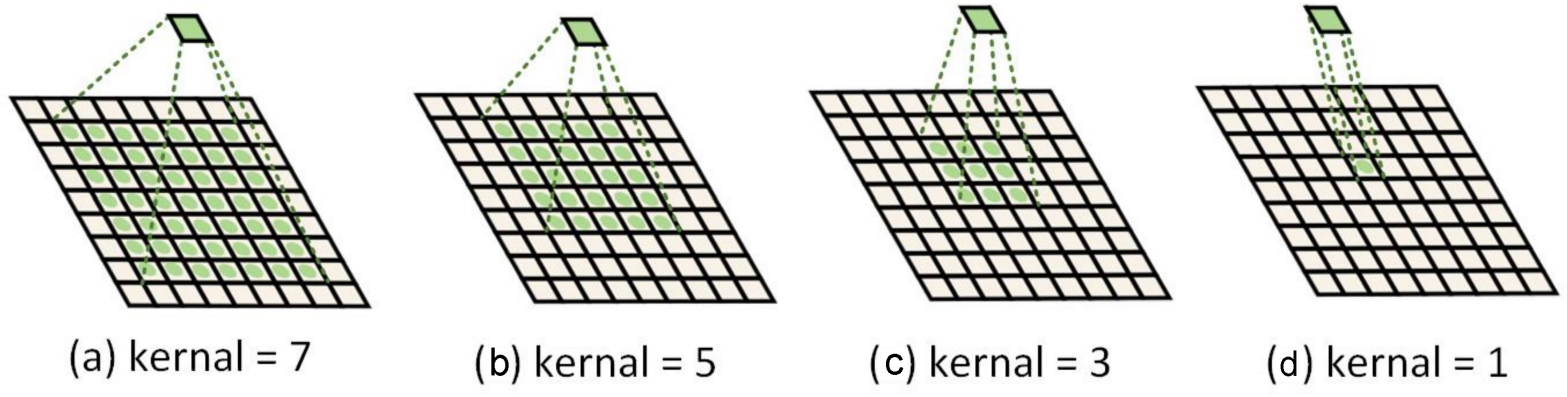
The different convolution kernel sizes and the corresponding receptive fields.

In order to analyze the impact of convolutional kernel size on classification accuracy, we separately changed the convolutional kernels in large-scale, medium-scale, and small-scale residual networks for classification. Large convolutional kernels have a larger receptive field and are better able to extract overall information from features. The smaller the convolutional kernel, the smaller the receptive field, which is more conducive to extracting detailed information from features. This is shown in Figure 15. The results in Table 3 compare the effects of different convolution kernel sizes on the results. When the main branches used 3 × 3 convolutional kernels and the residual branches used 1 × 1 convolutional kernels, the classification accuracy was the lowest, at only 93.9%. This is because small convolutional kernels cannot effectively extract global and texture information from features. When the main branch of the large-scale residual network used 5 × 5 convolutional kernels and the residual branch used 3 × 3 convolutional kernels, the classification accuracy improved to 94.6%. However, the extraction of global features cannot achieve optimal results. When the large-scale residual network used 7 × 7 and 5 × 5 convolutional kernels, and the medium-scale residual network used 5 × 5 and 3 × 3 convolutional kernels, the classification accuracy was the highest, reaching 96.9%. The results indicate that the model effectively extracted global information, texture information, and semantic information.

### 5.9. The Impact of TC Weighting Mechanism on Classification Accuracy

The TC weighting mechanism consists of time weighting and channel weighting. It can allocate weights based on time segments and channel importance, effectively extracting important feature information. In Table 4, the effects of time weighting and channel weighting on the results are compared. When no weights were added to the network, the classification accuracy was only 92.5%. This classification had the lowest accuracy. The accuracy of the network structure with time weighting was 94.6%. When only adding channel weighting, the classification accuracy was 94.1%, indicating that time weighting is more effective for time–Doppler images classification. The complete MRW-CN network structure achieved an accuracy of 96.9% in classifying time–Doppler images. This indicates that the TC weighting mechanism is crucial for classification.

### 5.10. Ablation Study

As shown in Table 5, we also performed ablation experiments to demonstrate that MRW-CN is an overall effective framework. The results demonstrate the effectiveness and necessity of residual branching and the time–channel weighting mechanism of different residual networks to improve the performance of HAR in the MRW image encoder.

To demonstrate the necessity and effectiveness of the components in the model, some ablation studies were conducted on the performance of active classification using our dataset. We investigated the contributions of the 5 × 5 convolutional kernel residual branches and the time–channel weighting mechanism in the large-scale residual network, the 3 × 3 convolutional kernel residual branch and the time–channel weighting mechanism in the medium-scale residual network, and the 1 × 1 convolutional kernel residual branch and the time–channel weighting mechanism in the small-scale residual network, respectively.

The first line represents the maximum classification accuracy of 96.9% obtained by retaining the six components. Comparing the first to third rows, we find that removing the 1 × 1 convolutional kernel residual branch and the time–channel weighting mechanism in the small convolutional kernel residual network reduced the classification accuracy by 0.8% and 2.5%, respectively. Comparing the third to fifth rows, removing the 3 × 3 convolutional kernel residual branch in the medium-scale residual network reduced the accuracy by 1.4%, and after removing the time–channel weighting mechanism, the classification accuracy was reduced by 0.3%. Comparing lines five through seven, removing the 5 × 5 convolutional kernel residual branch in the large-scale residual network, the accuracy only decreased by 1.1%, and the classification accuracy dropped to 89.1% after removing the time–channel weighting mechanism. It shows that each part contributed to the classification accuracy. And since the detailed information was extracted from the small-scale network, and the detailed information included a large number of feature representations in the activity, it had the greatest impact on the classification results. The global information contained fewer moderately important features, and the global information extracted from the modules in the large-scale residual network also had the least impact on classification accuracy.

Table 3 indicates that each of the six components in the MRW-CN model is indispensable. When combined together, they help significantly improve model performance.

## 6. Conclusions

In this paper, we propose the MRW-CN to achieve human activity recognition. Firstly, the proposed MRW image encoder performs potential feature representation vectors on unlabeled radar time–Doppler images through contrastive learning. This process involves the utilization of the large-scale residual networks to extract broader global information, the medium-scale residual networks to capture deeper texture information, and the small-scale residual networks to extract finer semantic information. The time–channel weighting mechanisms can focus on more important time periods and channel layers. After that, the downstream classifier can effectively classify human activities with a small amount of labeled data for training. The proposed MRW-CN method was evaluated using our measured radar-based human activity recognition (HAR) dataset that encompasses eight human activities. The experimental results demonstrated the effectiveness of the method, with a max accuracy of 96.9% achieved when only 10% of the labeled data were used. Comparative experiments also showed the impact of batch sizes and the parameter τ on recognition accuracy. Ablation experiments analyzed the joint effect of the residual network of different convolutional kernels and the time–channel weighting mechanism on recognition accuracy, showing the effectiveness of each constituent module. We also analyzed the synergy of the model components through feature visualization. The MRW-CN model achieved a recognition accuracy of 97.6% when fully supervised training was performed. In order to have a wider range of applications in practical situations, we will add more classes of activities in data collection in the future, such as “running” and “chopping”. We will also incorporate more data collection scenarios, such as in classrooms and vehicles. By combining the extraction of Doppler information by radar and the effective extraction of spatial information of objects by cameras, complementary advantages can be achieved. In the future, we will use radar and camera fusion algorithms to classify more classes of activities in more scenarios.

## Figures and Tables

**Table 1:** The different types of datasets collected by different sensors.

	Sensors	Data Type
non-invasive	accelerometer	time series (one-dimensional data)
	gyroscope	
	IMUs	
	magnetometers	
	electrodes (EEGs)	
	electrodes (sEMGs)	
	radar	images (two-dimensional data)
invasive	electrodes (iEMG)	time series (one-dimensional data)

**Table 2:** Performance comparison of different methods.

Methods	Labeled Data		FLOPs	Inference Time (10% Labeled Data)
	5%	10%		
SSTL [51]	85.5 ± 2.9	86.2 ± 3.0	4.1 G	3.1 h
SimCLR [45]	88.8 ± 0.4	89.8 ± 0.7	3.9 G	3.5 h
BYOL [46]	90.6 ± 1.2	91.0 ± 0.5	4.1 G	2.8 h
JDS-TL [38]	91.5 ± 0.9	92.9 ± 0.3	4.0 G	2.9 h
MOCO [52]	92.6 ± 1.1	93.1 ± 0.5	4.2 G	3 h
SelfHAR [53]	93.6 ± 1.2	95.1 ± 0.4	4.1 G	2.9 h
**MRW-CN**	**94.7 ± 0.4**	**96.3 ± 0.6**	3.8 G	3 h

**Table 3:** The influence of convolutional kernel size on classification accuracy.

	Large		Medium		Small		Max Accuracy (%)
	Main Branch	Residual Branch	Main Branch	Residual Branch	Main Branch	Residual Branch	
(1)	3	1	3	1	3	1	93.9
(2)	5	3	3	1	3	1	94.6
(3)	5	3	5	3	3	1	95.8
(4)	7	5	5	3	3	1	96.9

**Table 4:** The impact of the TC weighting mechanism on classification accuracy.

	Time Weight	Channel Weight	Max Accuracy (%)
(1)	×	×	92.5
(2)	✔	×	94.6
(3)	×	✔	94.1
(4)	✔	✔	96.9

**Table 5:** Ablation study on MRW-CN.

	Large		Medium		Small		Max Accuracy (%)
	TC Weight	con5	TC Weight	con3	TC Weight	con1	
(1)	✔	✔	✔	✔	✔	✔	96.9
(2)	✔	✔	✔	✔	✔	×	95.8
(3)	✔	✔	✔	✔	×	×	94.4
(4)	✔	✔	✔	×	×	×	94.1
(5)	✔	✔	×	×	×	×	92.9
(6)	✔	×	×	×	×	×	91.8
(7)	×	×	×	×	×	×	89.1

## Data Availability

Data are contained within the article.

## References

[1] Qiao X., Amin M.G., Shan T., Zeng Z., Tao R. (2021). Human activity classification based on micro-Doppler signatures separation. IEEE Trans. Geosci. Remote Sens..

[2] Jokanović B., Amin M. (2017). Fall detection using deep learning in range-Doppler radars. IEEE Trans. Aerosp. Electron. Syst..

[3] Severino J.V.B., Zimmer A., Brandmeier T., Freire R.Z. (2019). Pedestrian recognition using micro Doppler effects of radar signals based on machine learning and multi-objective optimization. Expert Syst. Appl..

[4] Chakraborty M., Kumawat H.C., Dhavale S.V., Raj A.A.B. (2022). DIAT-*μ* RadHAR (micro-Doppler signature dataset) & *μ* RadNet (a lightweight DCNN)—For human suspicious activity recognition. IEEE Sens. J..

[5] Mishra A., Sharma S., Kumar S., Ranjan P., Ujlayan A. (2021). Effect of hand grip actions on object recognition process: A machine learning-based approach for improved motor rehabilitation. Neural Comput. Appl..

[6] AlMuhaideb S., AlAbdulkarim L., AlShahrani D.M., AlDhubaib H., AlSadoun D.E. (2024). Achieving More with Less: A Lightweight Deep Learning Solution for Advanced Human Activity Recognition (HAR). Sensors.

[7] LeCun Y., Bengio Y., Hinton G. (2015). Deep learning. Nature.

[8] Semwal V.B., Gaud N., Lalwani P., Bijalwan V., Alok A.K. (2022). Pattern identification of different human joints for different human walking styles using inertial measurement unit (IMU) sensor. Artif. Intell. Rev..

[9] Challa S.K., Kumar A., Semwal V.B. (2022). A multibranch CNN-BiLSTM model for human activity recognition using wearable sensor data. Vis. Comput..

[10] Dua N., Singh S.N., Semwal V.B. (2021). Multi-input CNN-GRU based human activity recognition using wearable sensors. Computing.

[11] Abdel-Salam R., Mostafa R., Hadhood M. (2021). Human activity recognition using wearable sensors: Review, challenges, evaluation benchmark. International Workshop on Deep Learning for Human Activity Recognition, Proceedings of the Conjunction with IJCAI-PRICAI 2020, Kyoto, Japan, 8 January 2021.

[12] Dua N., Singh S.N., Semwal V.B., Challa S.K. (2023). Inception inspired CNN-GRU hybrid network for human activity recognition. Multimed. Tools Appl..

[13] Salehzadeh A., Calitz A.P., Greyling J. (2020). Human activity recognition using deep electroencephalography learning. Biomed. Signal Process. Control.

[14] Rani G.J., Hashmi M.F., Gupta A. (2023). Surface electromyography and artificial intelligence for human activity recognition-A systematic review on methods, emerging trends applications, challenges, and future implementation. IEEE Access.

[15] Challa S.K., Kumar A., Semwal V.B., Dua N. (2022). An optimized-LSTM and RGB-D sensor-based human gait trajectory generator for bipedal robot walking. IEEE Sens. J..

[16] Patil P., Kumar K.S., Gaud N., Semwal V.B. (2019). Clinical human gait classification: Extreme learning machine approach. Proceedings of the 2019 1st International Conference on Advances in Science, Engineering and Robotics Technology (ICASERT).

[17] Semwal V.B., Jain R., Maheshwari P., Khatwani S. (2023). Gait reference trajectory generation at different walking speeds using LSTM and CNN. Multimed. Tools Appl..

[18] Zhang Y., Tang H., Wu Y., Wang B., Yang D. (2024). FMCW Radar Human Action Recognition Based on Asymmetric Convolutional Residual Blocks. Sensors.

[19] Chen V.C. (2003). Micro-Doppler effect of micromotion dynamics: A review. Indep. Compon. Anal. Wavelets Neural Netw..

[20] Xiong Z., Zhang J., Yin J., Xiong G. UWB Radar Traffic gesture recognition Based on Range-Doppler Dual-Channel Fusion Visual Transformer Network. Proceedings of the 2024 8th International Conference on Digital Signal Processing.

[21] Diaz G., Tan B., Sobron I., Eizmendi I., Landa I., Velez M. (2024). Cross-Domain Human Activity Recognition Using Low-Resolution Infrared Sensors. Sensors.

[22] Wang R., Ren J., Li W., Yu T., Zhang F., Wang J. (2024). Application of Instance Segmentation to Identifying Insect Concentrations in Data from an Entomological Radar. Remote Sens..

[23] Abdelrazik M.A., Zekry A., Mohamed W.A. (2023). Efficient Hybrid Algorithm for Human Action Recognition. J. Image Graph..

[24] He Y., Li X., Jing X. (2019). A mutiscale residual attention network for multitask learning of human activity using radar micro-Doppler signatures. Remote Sens..

[25] Liu X., Zhang F., Hou Z., Mian L., Wang Z., Zhang J., Tang J. (2021). Self-supervised learning: Generative or contrastive. IEEE Trans. Knowl. Data Eng..

[26] Liu R. (2021). Understand and improve contrastive learning methods for visual representation: A review. arXiv.

[27] Kumar P., Rawat P., Chauhan S. (2022). Contrastive self-supervised learning: Review, progress, challenges and future research directions. Int. J. Multimed. Inf. Retr..

[28] Ding X., Zhang X., Han J., Ding G. Scaling up your kernels to 31 × 31: Revisiting large kernel design in cnns. Proceedings of the IEEE/CVF Conference on Computer Vision and Pattern Recognition.

[29] Brendel W., Bethge M. (2019). Approximating cnns with bag-of-local-features models works surprisingly well on imagenet. arXiv.

[30] Wang X., Hui B., Guo P., Jin R., Ding L. (2024). Coarse-to-Fine Structure and Semantic Learning for Single-Sample SAR Image Generation. Remote Sens..

[31] Gurbuz S.Z., Rahman M.M., Kurtoglu E., Macks T., Fioranelli F. (2020). Cross-frequency training with adversarial learning for radar micro-Doppler signature classification (Rising Researcher). Proceedings of the Radar Sensor Technology XXIV.

[32] Yang Y., Hou C., Lang Y., Sakamoto T., He Y., Xiang W. (2019). Omnidirectional motion classification with monostatic radar system using micro-Doppler signatures. IEEE Trans. Geosci. Remote Sens..

[33] Kim Y., Ling H. (2009). Through-wall human tracking with multiple Doppler sensors using an artificial neural network. IEEE Trans. Antennas Propag..

[34] Erol B., Gurbuz S.Z., Amin M.G. (2020). Motion classification using kinematically sifted acgan-synthesized radar micro-doppler signatures. IEEE Trans. Aerosp. Electron. Syst..

[35] Seyfioglu M., Gurbuz S. (2018). Deep convolutional autoencoder for radar-based human activity recognition. IEEE Trans. Aerosp. Electron. Syst..

[36] Park J., Javier R.J., Moon T., Kim Y. (2016). Micro-Doppler based classification of human aquatic activities via transfer learning of convolutional neural networks. Sensors.

[37] Taylor W., Dashtipour K., Shah S.A., Hussain A., Abbasi Q.H., Imran M.A. (2021). Radar sensing for activity classification in elderly people exploiting micro-doppler signatures using machine learning. Sensors.

[38] Li X., He Y., Fioranelli F., Jing X. (2021). Semisupervised human activity recognition with radar micro-Doppler signatures. IEEE Trans. Geosci. Remote Sens..

[39] Du H., He Y., Jin T. (2018). Transfer learning for human activities classification using micro-Doppler spectrograms. Proceedings of the 2018 IEEE International Conference on Computational Electromagnetics (ICCEM).

[40] Hadsell R., Chopra S., LeCun Y. (2006). Dimensionality reduction by learning an invariant mapping. Proceedings of the 2006 IEEE Computer Society Conference on Computer Vision and Pattern Recognition (CVPR’06).

[41] Dosovitskiy A., Springenberg J.T., Riedmiller M., Brox T. Discriminative unsupervised feature learning with convolutional neural networks. Proceedings of the 27th International Conference on Neural Information Processing Systems.

[42] Eldele E., Ragab M., Chen Z., Wu M., Kwoh C.K., Li X., Guan C. (2022). Self-supervised contrastive representation learning for semi-supervised time-series classification. arXiv.

[43] Chen D., Chen Y., Li Y., Mao F., He Y., Xue H. (2021). Self-supervised learning for few-shot image classification. Proceedings of the ICASSP 2021–2021 IEEE International Conference on Acoustics, Speech and Signal Processing (ICASSP).

[44] Sarkar P., Etemad A. (2020). Self-supervised ECG representation learning for emotion recognition. IEEE Trans. Affect. Comput..

[45] Chen T., Kornblith S., Norouzi M., Hinton G. A simple framework for contrastive learning of visual representations. Proceedings of the International Conference on Machine Learning. PMLR.

[46] Grill J.B., Strub F., Altché F., Tallec C., Richemond P., Buchatskaya E., Doersch C., Avila Pires B., Guo Z., Gheshlaghi Azar M. (2020). Bootstrap your own latent-a new approach to self-supervised learning. Adv. Neural Inf. Process. Syst..

[47] Chen X., He K. Exploring simple siamese representation learning. Proceedings of the IEEE/CVF Conference on Computer Vision and Pattern Recognition.

[48] Chen T., Kornblith S., Swersky K., Norouzi M., Hinton G.E. (2020). Big self-supervised models are strong semi-supervised learners. Adv. Neural Inf. Process. Syst..

[49] Tang C.I., Perez-Pozuelo I., Spathis D., Mascolo C. (2020). Exploring contrastive learning in human activity recognition for healthcare. arXiv.

[50] Elfwing S., Uchibe E., Doya K. (2018). Sigmoid-weighted linear units for neural network function approximation in reinforcement learning. Neural Netw..

[51] Gupta R., Sahu S., Espy-Wilson C., Narayanan S. (2018). Semi-supervised and transfer learning approaches for low resource sentiment classification. Proceedings of the 2018 IEEE International Conference on Acoustics, Speech and Signal Processing (ICASSP).

[52] He K., Fan H., Wu Y., Xie S., Girshick R. Momentum contrast for unsupervised visual representation learning. Proceedings of the IEEE/CVF Conference on Computer Vision and Pattern Recognition.

[53] Tang C.I., Perez-Pozuelo I., Spathis D., Brage S., Wareham N., Mascolo C. (2021). SelfHAR: Improving Human Activity Recognition through Self-training with Unlabeled Data. Proc. ACM Interact. Mob. Wearable Ubiquitous Technol..

